# Cow's Milk and Rickets

**Published:** 1908-05-30

**Authors:** G. Arbour Stephens

**Affiliations:** 61 Walter Road, Swansea


### Editor's Letter-Box.

### COW'S MILK AND RICKETS.

Sir,—The point I raised and endeavoured to emphasise in my first letter was the fact that about 90 per cent. of the milk consumed in this country was obtained from cows in calf. This fact I contend is sufficient to account for the inability of a large number of infants to digest ordinary cow's milk, and is a *raison d'etre* for the very extensively advertised baby-foods. The progressively increasing demands of the embryonic calf have a marked deteriorating influence on the quality of the milk, which no amount of diluting, precipitating, or even "Wrighting" can compensate.

The anonymous gibes of a "J" are about as valueless as his invocation of the Harley Street pantheon. I may have said somebody is a panacea, but I did not say somebody else displays opsomaniac tendencies! In concluding my contribution to this "little discussion" I make so bold as to prophesy that in five years' time "opsonin" as a technical term will be obsolete.

Yours truly,

G. Arbour Stephens.

61 Walter Road, Swansea, May 23, 1908.

THE HOSPITAL *May* 30, 1908.

